# Editorial: Quality of Life and Side Effects Management in Lung Cancer Treatment

**DOI:** 10.3389/fonc.2021.651797

**Published:** 2021-03-05

**Authors:** Alex Molassiotis, Patsy Yates, Janelle Yorke

**Affiliations:** ^1^School of Nursing, The Hong Kong Polytechnic University, Kowloon, Hong Kong; ^2^Faculty of Health, Queensland University of Technology, Brisbane, QLD, Australia; ^3^The Christie National Health Service (NHS) Foundation Trust, University of Manchester, Manchester, United Kingdom

**Keywords:** dyspnea, symptom management, quality of life, symptom cluster, research priorities, lung cancer—diagnosis

Despite significant advances in the treatment of lung cancer, particularly with the evolution of immunotherapies and targeted therapies, the symptom burden is still significant and impact on quality of life for these patients. One-third of lung cancer patients experienced moderate-to-severe symptoms in one study before initial treatment (1), with significantly higher incidence after treatment and as disease progresses (2). The symptom burden in lung cancer patients is, for many symptoms, higher than in other cancer diagnostic groups, as shown in an observational study of more than 120,000 cancer patients (2). Most frequent symptoms include fatigue, pain, psychological distress, breathlessness and cough, while histology and cancer stage differentially affect those symptoms, further affecting multiple domains of quality of life (1, 2). In advanced-stage lung cancer patients, high symptom burden has been significantly associated with overall survival, progression free survival, and objective response rate (3).

It is clear that managing symptoms related to lung cancer and its treatments can positively impact on both quality and quantity of life. Hence, in this Research Topic, we have put together six articles that deal with supportive care topics in the lung cancer population. Half of them focus on breathlessness. Sardaro et al. assessed 80 patients in a prospective study after radiation therapy over a period of 6 months. Their focus was on radiation-induced lung injury (an under-researched topic) and used dyspnea as symptomatic endpoint for lung injury. Parameters of lung volume-dose were strongly correlated with dyspnea, with an increase of 10%. The authors recommended regular assessment of dyspnea to identify early radiation-induced toxicity in the lungs.

The other two studies on breathlessness were randomized trials of non-pharmacological interventions. In a multicenter trial of 144 patients, Yates et al. showed that breathing exercises alongside psychosocial support over 8 weeks was significantly improved in the intervention group in relation to average breathlessness, control over breathlessness and anxiety scores. All these outcomes, however, were secondary outcomes. The primary outcome of “worst” dyspnea, and secondary outcomes for distress from breathlessness, functional status, and depression, did not show an improvement in the intervention group over the control group. This study adds to the limited pool of effective interventions we have to manage breathlessness, and to the consistent literature that breathing retraining and exercises with or without other intervention components can improve this symptom experience. The second trial by Choratas et al. was a small-size feasibility trial of 24 lung cancer patients and 24 of their informal caregivers. The intervention was educational in nature, focusing on teaching patients on aspects of breathlessness and introducing through video clips three interventions, including diaphragmatic breathing, inspiratory muscle training, and the use of a handheld fan. The trial showed that the design of this educational intervention is feasible and suggested improvements in both breathlessness levels and anxiety. However, despite showing some potential effects, a large-scale fully-powered trial is necessary to clarify the effectiveness of this intervention.

The study by Li et al. investigated the experience of symptoms in lung cancer patients receiving chemotherapy in a longitudinal design from before treatment to cycle one to cycle three and above, using latent profile analysis. Two symptom profiles were identified, including those patients with a “Mild” symptom profile and those with “Moderate-severe” one. During the time of assessment, about 41% of those in the mild group moved to have moderate-severe symptoms, whereas only 2% of the latter group moved to the mild symptom group. Eight symptom transition patterns were observed. This study highlights the changing nature of the symptom experience in this group of patients that needs to be considered in the development of interventions to manage the symptom experience.

Another study by Zhang et al. used data from 545 patients to develop a survival prognostic model. The best fit for the model (area under the curve = 0.73) included the following variables: age >/=65, TNM stage, lung lobectomy, chemotherapy type and pretreatment hemoglobin levels. The lack of a validation cohort in this study and its retrospective nature are major limitations but if this model is confirmed in future studies it can provide an easily-calculated prediction of survival in non-small cell lung cancer patients.

Some of our observations during the time we edited this Research Topic were about the poor quality of the submitted studies, limited interest in the topic and the narrow focus of studies. The final article of the Research Topic by Molassiotis et al. highlights research priorities in the field from the perspective of nurses and allied health professionals through an international online survey. The development of interventions, particularly around symptom management, were among the most frequently reported priorities, alongside interventions to improve quality of life and healthcare system issues (such as continuity of care or access to care). The list of priorities may be used by funders to stimulate specific research were there are gaps in the evidence and allow for urgently-needed evidence to be developed and subsequently used to improve the care of patients with lung cancer.

There is a need for more and better quality studies in the field of quality of life and symptom management in lung cancer. While lung cancer patients will live longer with the recent advances in treatments, the majority will still die from the disease, making quality of life, palliative care and end of life care significant priorities, requiring proactive assessment and management of symptoms, and a multidisciplinary care approach.

## Author Contributions

AM compiled the first draft. PY and JY commented on the draft and had intellectual input. All authors contributed to the article and approved the submitted version.

## Conflict of Interest

The authors declare that the research was conducted in the absence of any commercial or financial relationships that could be construed as a potential conflict of interest.
